# The Effects of Bairesi Complex Prescription (a Uyghur Medicine Prescription) and Its Five Crude Herbal Extracts on Melanogenesis in G-361 Cells

**DOI:** 10.1155/2016/8415359

**Published:** 2016-03-16

**Authors:** Xuedan Huang, Masayuki Ishikawa, Arkin Mansur, Aynur Emet, Ezimet Nasir, Repket Semet, Yoshinori Kobayashi

**Affiliations:** ^1^School of Pharmaceutical Sciences, Kitasato University, 5-9-1 Shirokane, Minato-ku, Tokyo 108-0086, Japan; ^2^Department of Science, Uyghur Medicine Hospital, Urumqi 830049, China

## Abstract

Vitiligo is considered a preimmune stage of a disease that is not well clarified. This condition is difficult to treat because there is no definite cure. Uyghur medicine is an important part of traditional Chinese medicine. There are many types of prescriptions that are used for the treatment of vitiligo. Bairesi complex prescription is one of the active prescriptions for vitiligo that is used in the clinic. However, the intensities of melanogenesis due to uses of Bairesi complex prescription and its five constituent crude herbs have not been reported yet. In the present study, we found that the hot water extracts of Bairesi complex prescription and the crude herbs were more effective in eliciting melanin production in G-361 cells than the EtOH extracts. Furthermore, the Bairesi complex prescription exhibited less cytotoxicity and was more effective in melanin formation than the five crude herbal extracts. In the present study, we also discuss the mechanisms of melanogenesis due to the use of the Bairesi complex prescription and its single crude herbal extracts.

## 1. Introduction

Vitiligo is a depigmentation disorder that causes the appearance of white spots on the skin due to the loss of the functional melanocytes. Vitiligo affects 1% of the world population, but the prevalence has been reported to be as high as 4% in South Asian, Mexican, and American populations. In most cases, vitiligo develops early in life between the ages of 10 and 30 years. Men and women are equally likely to develop vitiligo [1].

The pathway of melanocyte loss in vitiligo is thought to be a complex interaction between genetic, environmental, biochemical, and immunological events, but this pathway is not well clarified. Oxidative stress is one possible pathogenic event in vitiligo [1, 2]. Defective recycling of tetrahydrobiopterin in the whole epidermises of patients with vitiligo is related to the intracellular production of reactive oxygen species (ROS) [3, 4]. The role of ROS in melanin formation is dual and complex. ROS can function as inhibitors of tyrosinase, and in the presence of H2O2, DOPA (dihydroxyphenylalanine) substrate, ROS can generate secondary complexes that can bind to and inhibit tyrosinase [5]. On the other hand, ROS also accelerate melanin formation from DOPA and other melanin intermediates without any enzymatic activity. Further, increased ROS levels in melanocytes may cause defective apoptosis that results in the release of aberrant proteins that can act as autoantigens that lead to autoimmunity [6]. ROS levels increase in response to cytokines, such as TNF-*α* (tumor necrosis factor-*α*) and TGF-*β*1 (transforming growth factor-*β*1), which are potent inhibitors of melanogenesis [7, 8]. 

The treatment of vitiligo remains a challenge for dermatologists. Although there are many therapeutic approaches, vitiligo is difficult to treat because there is no definite cure [9]. Topical corticosteroids, ultraviolet light, excimer laser, and surgical therapy are usually used in clinic [10]. The application of topical steroids induces long term side effects, and ultraviolet light therapy causes skin cancer. Many clinical trials that have examined the use of natural health product interventions (e.g., vitamins, minerals, herbal medicines, and other supplements) for vitiligo have been published [11].

Central Asian traditional Uyghur medicine belongs to the family of Unani medical systems derived from the Greco-Arabic tradition. Uyghur medicine is also an important part of traditional Chinese medicine that has been developed for 2500 years [12]. Uyghur medicine considers the four bodily fluids, that is, Khan, Belghem, Sapra, and Savda [13]. More than 450 types of herbal medicines are commonly used in clinics. In our pervious study, we determined the melanogenesis effects of 12 prescriptions that are typically used in the treatment of vitiligo in Uyghur clinics. Our results revealed four prescriptions that enhanced melanin production in G-361 cells. The Bairesi complex prescription was one of these active prescriptions, but the mechanism of the action of this prescription is not clear [14].

The Bairesi complex prescription contains the following five crude herbs: ①* Psoralea corylifolia* (Fabaceae, Chinese name: Buguzhi), ②* Plumbago zeylanica* (Plumbaginaceae, Chinese name: Baidanhua), ③* Brassica juncea* (Brassicaceae, Chinese name: Huangjiezi), ④* Nigella glandulifera* (Ranunculaceae, Chinese name: Heizhongcaozi), and ⑤* Vernonia anthelmintica* (Asteraceae, Chinese name: Quchongbanjiuju). The effects of* P. corylifolia *and* V. anthelmintica* on melanogenesis have been reported in some studies [15, 16]. Psoralen is a light-sensitive drug contained in the seeds of* P. corylifolia*, which has been wildly used in psoralen plus ultraviolet light therapy (PUVA) treatments for vitiligo [17]. Although* P. corylifolia *and* V. anthelmintica* are constituents of the Bairesi complex prescription, the intensities of the effects of these plants on melanogenesis in isolation and in the Bairesi complex prescription remain unknown. In this study, we clarified the effects of each of the crude herbs and the Bairesi complex prescription on melanin production. Furthermore, we also compared the melanogenic effects of the water and ethanol extracts of the crude herbs and the Bairesi complex prescription.

## 2. Materials and Methods

### 2.1. Materials

2′,7′-Dichlorodihydrofluorescein-diacetate (DCFH-DA) was purchased from Sigma Chemical Co. Ltd. Antibodies against tyrosinase, TRP-1, and TRP-2 were purchased from R&D Systems, Inc., Abcam PLC, and Santa Cruz Biotechnology, Inc., respectively. Fetal bovine serum (FBS) was purchased from Life Technologies Inc. Gibco (Gibco BRL, Grand Island, NY, USA). The other chemicals used in this study were special grade commercial products.

### 2.2. Fractionation of the Crude Herbs and the Bairesi Complex Prescription

The crude herbs of the Bairesi complex prescription were purchased from the Herbal Pharmacy of the Uyghur Medicine Hospital. Each crude herb was extracted with ethanol (EtOH) or hot water as follows: (1) The crude herbs or an equal amount of the crude herbs mix (Bairesi complex prescription, 5 g) was extracted with EtOH for 4 h with sonication, and the solution was then filtered and evaporated at 40°C* in vacuo* until dry. The yields of* P. corylifolia*,* P. zeylanica*,* B. juncea*,* N. glandulifera*,* V. anthelmintica*, and the crude herbs mix were 1.24, 0.59, 0.61, 1.15, 0.84, and 1.02 g, respectively. (2) The crude herbs or an equal amount of the crude herbs mix (5 g) was extracted with hot water (100°C) for 1 h, and the solution was then freeze-dried. The yields of* P. corylifolia*,* P. zeylanica*,* B. juncea*,* N. glandulifera*,* V. anthelmintica * and the crude herbs mix were 0.52, 0.51, 0.64, 0.42, 0.52, and 0.62 g, respectively.

### 2.3. Cell Line and Culture

The G-361 human skin melanoma cells were purchased from the Health Science Research Resources Bank (Japan Health Sciences Foundation, cell number: IF05009) and cultured in Dulbecco's modified Eagle's medium supplemented with 10% FBS in humidified incubator containing 5% CO_2_ in air at 37°C. The cells were treated with the test sample for 24 h after seeding. All the test samples were dissolved in dimethyl sulfoxide (DMSO). They were diluted in the culture medium before being used (final DMSO concentration is 0.1%). Final concentration of each sample was described in following experiments.

Methoxsalen (Sigma Japan), a drug of melanin production used to treat vitiligo, was used as a positive control for melanogenesis.

### 2.4. Cell Viability Assay

The cell viability was measured by MTT assay, which is based on the conversion of MTT to formazan crystals by mitochondrial dehydrogenases [18]. Briefly, the G-361 cells were incubated at a density of 2 × 10^4^ cells/well in 96-well plates. After 24 h, each of the samples or methoxsalen (0.1~100 *μ*M) was added to the cells and incubated for 72 h. At the end of treatment, 10 *μ*L of 5 mg/mL MTT was added to each well and incubated for 4 h at 37°C. After the removal of the culture medium, 200 *μ*L of DMSO was added to each well to dissolve the formazan formed in the reaction. The absorbance of each well was measured at 540 nm using a microplate reader (Bio Rad, model 680). The viability is expressed as (*A*_540_-treated cells/*A*_540_ of appropriate control) × 100% after correction for the background absorbance (100% cytotoxicity).

### 2.5. Determination of the Melanin Content

This assay followed Ando's method [19] with slight modification. Briefly, subcultures of G-361 cells were seeded in 96-well plates at a density of 5 × 10^4^ cells/mL and cultured for 24 h. The medium was then replaced with 200 *μ*L of fresh 10% FBS-DMEM and the sample or methoxsalen (0.1~100 *μ*M). After culturing for 3 days, the cells were harvested and suspended in 50 *μ*L of a 1 N NaOH-10% DMSO solution (v/v) and maintained at 80°C for 1 h. The melanin content was then determined by reading the absorbance at 405 nm. The control cells were cultured in a medium containing 0.1% DMSO without any samples.

### 2.6. DOPA Staining

After treatment with the samples for 72 h, the cells were washed twice in PBS. Next, the cells were fixed with 10% formalin solution (Wako) for 10 mins, washed with PBS, and incubated in 0.1 M sodium phosphate buffer (pH 7.4) containing 0.1% DOPA (L-3,4-dihydroxyphenylalanine) at 37°C in the dark. After 4 h, the solution was removed, and the cells were observed with phase contrast microscopy (Nikon).

### 2.7. Western Blotting

The cells were dissolved for 30 mins with lysis buffer (150 mM NaCl, 50 mM tris (pH 7.2), 1 mM EDTA, 0.5% sodium deoxycholate, 1% Nonidet P-40, 1 mM sodium vanadate, 1 mM NaF, 20 *μ*g/mL aprotinin, 50 *μ*g/mL leupeptin, 10 *μ*g/mL pepstatin A, and 100 *μ*g/mL phenylmethylsulfonyl fluoride) after the treatment of the samples for 72 h. Next, the solution was centrifuged at 2000 ×g for 20 mins at 4°C. The supernatants were collected, and the protein concentrations were determined with a BCA protein assay kit (Thermo). Equal amounts of protein were fractionated on 10% SDS-PAGE gels and transferred to 0.45 *μ*m PVDF (Hybond; Amersham Pharmacia Biotech). After blocking overnight in 0.1% Tween-20 and 5% nonfat dry milk in PBS, the blots were incubated with antibody for 1 h at room temperature. After washing, the membrane was reincubated with 1 : 750 diluted biotinylated mouse IgG or rabbit IgG for 1 h at room temperature. The membrane was washed several times and incubated with 1 : 750 diluted horseradish peroxidase-coupled streptavidin for 1 h at room temperature. After several washing steps, the color reaction was developed with tetramethylbenzidine (TMB, Sigma). Densitometry analyses of the protein bands were performed with the software Scion Image.

### 2.8. ROS Production Assay

DCFH-DA is a relatively specific probe for hydrogen peroxide and was used to study the intracellular ROS formation. The cells were treated with hot water extracts (62.5 *μ*g/mL) for 6 h, 24 h, 48 h, or 72 h and then incubated with 5 *μ*M DCFH-DA at 37°C for 15 min. The DCF fluorescence was measured using a flow cytometer at excitation and emission wavelengths of 488 and 525 nm, respectively.

### 2.9. Statistical Analyses

The data are represented as the means ± the standard deviations of the means (SD). The significances of the differences in the assay values were evaluated with ANOVA followed by Tukey's multiple comparison tests. *p* < 0.05 was taken to indicate a statistically significant difference.

## 3. Results

### 3.1. Effects of the EtOH and Hot Water Extracts on Cell Viability

**Figure 1:**
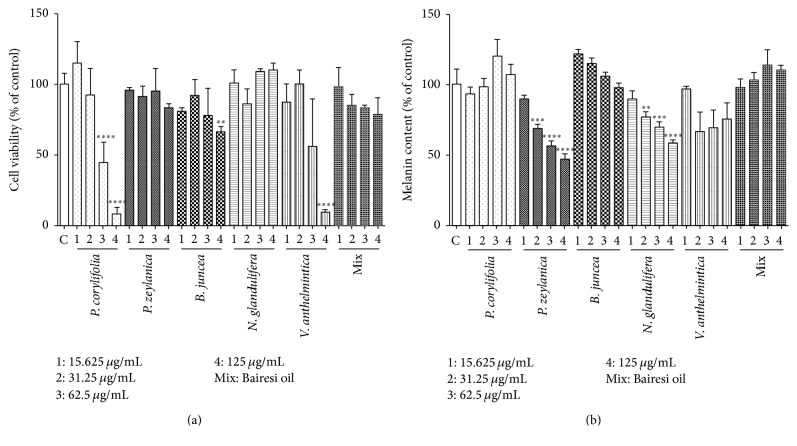
Effect of the EtOH extracts of the Bairesi complex prescription and the five crude herbs on cell viability and melanin content in G-361 cells. The cells were cultured in Dulbecco's modified Eagle's medium containing 10% FBS for three days and subsequently diluted and incubated again in fresh medium with or without a sample. Cell viabilities and melanin contents were measured 72 h later as described in Section 2. The results are representative of three separate determinations. Each point is the mean (±SD) of three experiments. ^*∗∗*^ *p* < 0.01, ^*∗∗∗*^ *p* < 0.001, ^*∗∗∗∗*^ *p* < 0.0001.

**Figure 2:**
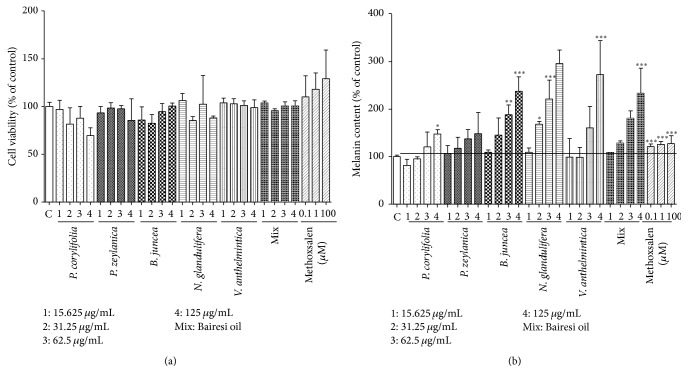
Effects of the water extracts of the Bairesi complex prescription and the five crude herbs on cell viabilities and melanin contents of the G-361 cells. The cells were cultured in Dulbecco's modified Eagle's medium containing 10% FBS for three days and subsequently diluted and incubated again in fresh medium with or without a sample. The cell viabilities or melanin contents were measured 72 h later as described in Section 2. The results are representative of three separate determinations. Each point is the mean (±SD) of three experiments. ^*∗*^ *p* < 0.05, ^*∗∗*^ *p* < 0.01, ^*∗∗∗*^ *p* < 0.001.

The effects of the EtOH and hot water extracts (15.625, 31.25, 62.5, and 125 *μ*g/mL) on cell viability were examined in the G-361 cells via the MTT method. As shown in Figure 1(a), the EtOH extracts of* P. corylifolia *(62.5~125 *μ*g/mL)*, B. juncea *(125 *μ*g/mL),and* V. anthelmintica* (125 *μ*g/mL) decreased cell viability. However, none of hot water extracts of the crude herbs (0~125 *μ*g/mL) affected the viabilities of the G-361 cells (Figure 2(a)). Methoxsalen (0.1–100 *μ*M) also did not affect cell viability (Figure 2(a)).

### 3.2. Effects of the EtOH and Hot Water Extracts on the Melanin Contents

The effects of the EtOH and hot water extracts (15.625, 31.25, 62.5, and 125 *μ*g/mL) on the melanin contents of the G-361 cells were examined. As shown in Figure 1(b), the EtOH extracts of* P. zeylanica *and* N. glandulifera *decreased the melanin contents in dose-dependent manners. In contrast, the* P. corylifolia, B. juncea, V. anthelmintica*, and mixed extracts (0–125 *μ*g/mL) did not affect the melanin contents. However, the hot water extracts of these plants and methoxsalen increased the melanin contents of the G-361 cells in dose-dependent manners (Figure 2(b)). The effective concentration (62.5 *μ*g/mL) of melanogenesis was used in the following experiments.

### 3.3. Effects of the Hot Water Extracts on DOPA Staining

**Figure 3:**
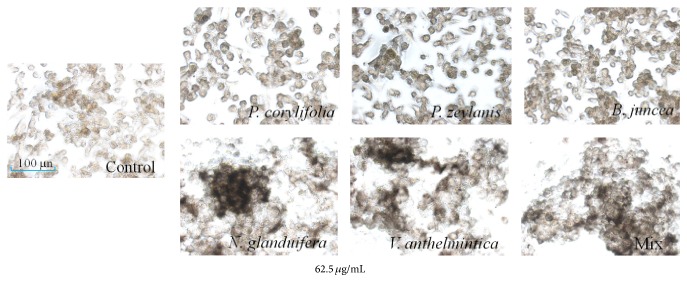
Effects of the Bairesi complex prescription and the crude herbal extracts on melanin formation activity in the G-361 cells. The cells were treated with or without the Bairesi complex prescription and the crude herbal extracts. Tyrosinase activities were measured 72 h later via DOPA staining. Each point is the mean (±SD) of three experiments.

We also determined the melanin content via DOPA staining. The melanin contents of the cells that were incubated with samples for 72 h are shown in Figure 3. All cells were darkly stained compared with the control cells.

### 3.4. Effects of the Hot Water Extracts on the TYR, TRP-1, and TRP-2 Proteins

**Figure 4:**
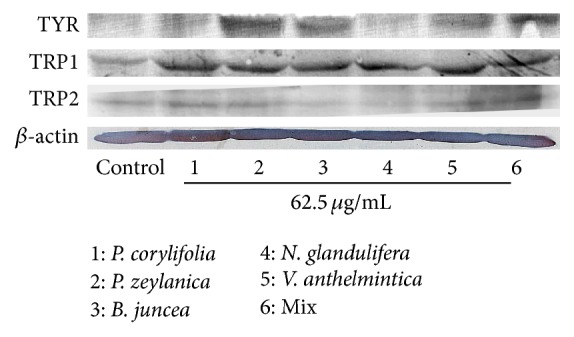
Effects of the Bairesi complex prescription and the crude herbal extracts on the TYR, TRP-1, and TRP-2 levels in the G-361 cells. The cells were incubated with the Bairesi complex prescription and the crude herbal extracts for 72 h. Cell lysis and western blotting were performed as described in Section 2. The data are representative of at least three independent experiments.

The effects of the hot water extracts on the TYR, TRP-1, and TRP-2 protein levels were measured via western blot analyses. As shown in Figure 4, TYR was increased by* P. zeylanica, B. juncea*, and* V. anthelmintica*. The TRP-1 levels increased in all of the cells that were treated with the samples for 72 h. However, the melanogenic effects of these drugs were not related to the TRP-2 protein.

### 3.5. Effects of the Hot Water Extracts on the Intracellular ROS Levels

**Figure 5:**
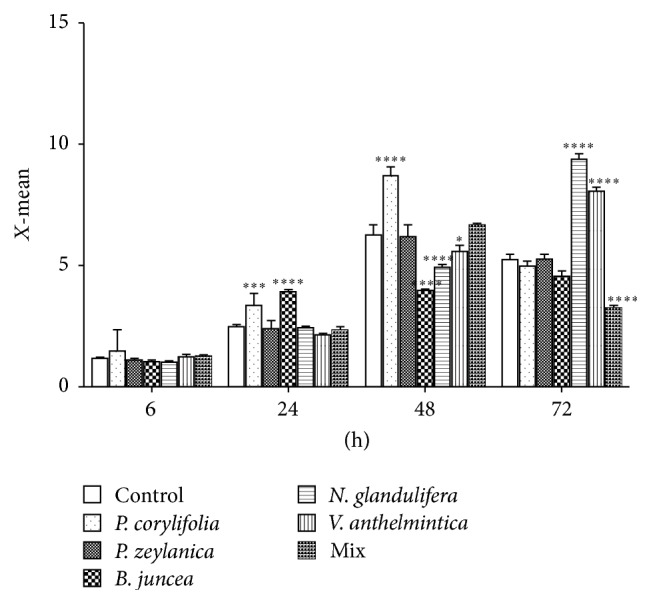
Effects of the Bairesi complex prescription and the crude herbal extracts on the intracellular ROS levels in the G-361 cells. The intracellular ROS levels were measured by flow cytometry. The DCF fluorescence intensities of the cells were measured at 6, 24, 48, and 72 h after treatment with 62.5 *μ*g/mL of the water extracts of the Bairesi complex prescription and the crude herbal extracts. The data are presented as the means ± the SD. ^*∗*^ *p* < 0.05, ^*∗∗∗*^ *p* < 0.001, ^*∗∗∗∗*^ *p* < 0.0001 compared to the control group at the same time points.

We examined the effects of the hot water extracts on the intracellular peroxides levels via flowcytometry. As shown in Figure 5, the intracellular ROS levels were not altered in any of the cells that were treated with the extracts for 6 h. The* P. corylifolia* extracts increased the ROS levels at 24 and 48 h. The* B. juncea* extract increased the ROS levels at 24 h but decreased these levels at 48 h. The* N. glandulifera* and* V. anthelmintica* extracts decreased the ROS levels at 48 h and increased them at 72 h. However, the* P. zeylanica* and mixed extracts did not increase the ROS levels at any time of treatment.

### 3.6. Effects of the Mix Extracts on Cell Viability and Melanin Content

**Figure 6:**
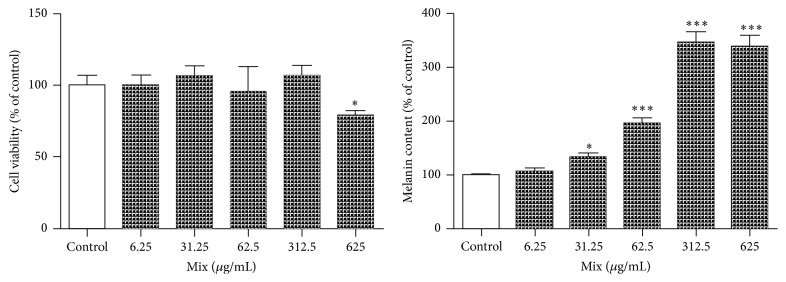
Effects of the mixed extracts on cell viabilities and melanin contents. The cells were cultured in Dulbecco's modified Eagle's medium containing 10% FBS for three days and subsequently diluted and incubated again in fresh medium with or without a sample. The cell viabilities and melanin contents were measured 72 h later as described in Section 2. The results are representative of three separate determinations. Each point is the mean (±SD) of three experiments. ^*∗*^ *p* < 0.05, ^*∗∗∗*^ *p* < 0.001.

The effects of the mixed extracts (6.25, 31.25, 62.5, 312.5, and 625 *μ*g/mL) on the cell viability and melanin content are illustrated in Figure 6. The melanin contents were increased by the treatments with mixed extracts in a dose-dependent manner without an effect on the cell viability until the concentration of 312.5 *μ*g/mL was reached. However, each of the crude herbal extracts decreased the cell viabilities at the concentration higher than 250 *μ*g/mL (date not shown). The cytotoxicities of each of the crude herbal extracts were 2.5-fold higher than that of the mixed extract. These results suggest that the mixed extract exhibited lower cytotoxicity and elicited greater melanin production than the single crude herbal extracts.

## 4. Discussion

In the present study, we observed differences in the melanogenic effects of the hot water extracts and EtOH extracts of the Bairesi complex prescription (i.e., the mixed extract) and its crude herb. As shown in Figures 1 and 2(b), the water extracts of mixed and crude herbs dose-dependently increased the melanin contents of the G-361 cells. The effective concentrations were 62.5 *μ*g/mL and 125 *μ*g/mL. However, the EtOH extracts of the Bairesi complex prescription and its crude herb did not elicit this effect in the G-361 cells, and the cytotoxicities of the EtOH extracts were greater than those of the hot water extracts (Figure 1(a)). These results established that the hot water extracts were more effective than the EtOH extracts. The yield of the water extracts were less than that of the EtOH extracts (shown in Materials and Methods), suggesting the hot water extracts show greater stimulation of melanin formation than EtOH extracts. As shown in Figure 3, the melanogenic effects of the crude herbal extracts and the mixed extracts were further demonstrated with DOPA staining. The effects of the* N. glandulifera, V. anthelmintica*, and mixed extracts on melanogenesis were remarkable.

Melanogenesis is regulated by three specific enzymes: TYR, TRP-1, and TRP-2 [20]. TYR is a copper-containing glycoprotein and a key enzyme in melanin synthesis that can catalyze three different reactions: the hydroxylation of tyrosine to 3,4-dihydroxyphenylalanine (DOPA), the oxidation of DOPA to dopaquinone, and the conversion of dopaquinone to dopachrome and subsequently to dihydroindolizine (DHI) or indole 5,6-quinone-2-carboxylic acid (DHICA) [21, 22]. TRP-1 and TRP-2 have functional roles in this biosynthesis pathway. TRP-1 catalyzes the oxidation of DHICA, and TRP-2 (dopachrome tautomerase) catalyzes the conversion of dopachrome to DHICA [23]. Our results revealed all of the crude herbs and mixed extracts increased TRP-1 in G-361 cells. Moreover, the* P. zeylanica, B. juncea, V. anthelmintica*, and mixed extracts increased the TYR levels. However, the melanogenic effects of these drugs were not related to the TRP-2 protein (Figure 4). Our results showed that the* P. zeylanica, B. juncea, V. anthelmintica*, and mixed extracts exhibited high levels of melanin-producing activity. These results suggest that the crude herbal extracts have different mechanism for melanin formation and should be investigated in the future.

The role of ROS in melanin formation is dual and complex. ROS might inhibit tyrosinase, but they are also able to accelerate melanin formation from DOPA and other melanin intermediates without any enzymatic activity. In the present study, we examined the effects of the mixed and crude herb extracts on ROS levels at 6, 24, 48, and 72 hours. As shown in Figure 4, only two samples, that is, the* P. zeylanica* and mixed extracts, did not elicit increase in the ROS levels at any treatment time point. Our results suggest that the* P. zeylanica* and mixed extracts effectively increased the melanin content while exhibiting limited toxicity.

We also determined the cytotoxicities of these samples to G-361 cells. As shown in Figure 6, the mixed extracts did not affect cell viability at concentration below 625 *μ*g/mL. However, all of the crude herbal extracts decreased cell viability following treatment with 250 *μ*g/mL (data not shown). Furthermore, the mixed extract increased the melanin contents at all of the concentrations (Figure 6), but the crude herbal extracts increased the melanin contents only at the concentration of 125 *μ*g/mL (Figure 2). Furthermore, we found that only the mixed extract exhibited positive activity as shown in Figures 3–6. These results suggest that the cytotoxicities of the mixed extracts were weaker, but the mixed extracts elicited greater melanogenesis than the single crude herb.

In conclusion, our study revealed that the Bairesi complex prescription, which is a Uyghur medicine, is a potent medicine for increasing melanin production. The hot water extracts were more effective than the EtOH extracts. Furthermore, the hot water extract of the Bairesi complex prescription exhibited lower cytotoxicities and greater efficacies in terms of increasing melanin content than the five crude herbal extracts. All of the crude herbs and mixed extracts increased TRP-1 in G-361 cells after treatment for 72 h. And TYR levels were increased by* P. zeylanica, B. juncea, V. anthelmintica*, and mixed extracts. Furthermore, only the Bairesi complex prescription and* P. zeylanica* extracts did not increase ROS levels at any time of treatment. Together, these results suggested that the mix is a promising prescription for treatment of vitiligo but need further investigation.
